# Human Herpesvirus-6 and -7 in the Brain Microenvironment of Persons with Neurological Pathology and Healthy People

**DOI:** 10.3390/ijms22052364

**Published:** 2021-02-27

**Authors:** Sandra Skuja, Simons Svirskis, Modra Murovska

**Affiliations:** 1 Institute of Anatomy and Anthropology, Rīga Stradiņš University, Kronvalda blvd 9, LV-1010 Rīga, Latvia; 2 Institute of Microbiology and Virology, Rīga Stradiņš University, Rātsupītes str. 5, LV-1067 Rīga, Latvia; ssvirskis@latnet.lv (S.S.); Modra.Murovska@rsu.lv (M.M.)

**Keywords:** frontal lobe, temporal lobe, human herpesvirus 6, human herpesvirus 7, immune response, PCR, immunohistochemistry

## Abstract

During persistent human beta-herpesvirus (HHV) infection, clinical manifestations may not appear. However, the lifelong influence of HHV is often associated with pathological changes in the central nervous system. Herein, we evaluated possible associations between immunoexpression of HHV-6, -7, and cellular immune response across different brain regions. The study aimed to explore HHV-6, -7 infection within the cortical lobes in cases of unspecified encephalopathy (UEP) and nonpathological conditions. We confirmed the presence of viral DNA by nPCR and viral antigens by immunohistochemistry. Overall, we have shown a significant increase (*p* < 0.001) of HHV antigen expression, especially HHV-7 in the temporal gray matter. Although HHV-infected neurons were found notably in the case of HHV-7, our observations suggest that higher (*p* < 0.001) cell tropism is associated with glial and endothelial cells in both UEP group and controls. HHV-6, predominantly detected in oligodendrocytes (*p* < 0.001), and HHV-7, predominantly detected in both astrocytes and oligodendrocytes (*p* < 0.001), exhibit varying effects on neural homeostasis. This indicates a high number (*p* < 0.001) of activated microglia observed in the temporal lobe in the UEP group. The question remains of whether human HHV contributes to neurological diseases or are markers for some aspect of the disease process.

## 1. Introduction

Both human herpesvirus-6 (HHV-6) and human herpesvirus-7 (HHV-7) belong to the *Betaherpesvirinae* subfamily [1,2]. The majority of the world’s population is exposed to beta-herpesviruses (HHV) at the time of early childhood. Primary HHV infection causes *Roseola infantum*, which rarely is severe, and infrequently is a fatal illness [3,4]. 

Human herpesviruses are capable of establishing lifelong persistence by an involvement of different stages of the viral life cycle. During persistent viral infection, clinical manifestations may frequently not even appear. However, the putative long term influence is associated with the central nervous system (CNS) diseases such as encephalitis, Alzheimer’s disease, multiple sclerosis, and has a role in tumorigenesis and mood disorders [5,6,7,8,9,10,11,12,13,14,15,16].

A wide range of endogenous and exogenous factors is implicated in the reactivation of the virus, such as drugs and immunosuppression [17,18]. Host symbiosis with HHV can be realized as 1) a latent phase when few viral genes are expressed, no virions and licensed DNA synthesis can be detected, or 2) a lytic phase when most viral genes are expressed, extracellular virions and unlicensed DNA are synthesized [19,20,21]. The host immune system may regulate these fate-decisions concerning patterns of viral persistence [22,23]. Microglial cells are the dominant immune system cells of the CNS, providing the defense against pathogens in case of injury or disease of the brain. [24,25,26].

After crossing the body mucosal lining, HHV-6, -7 can enter potential replication compartments in sensory ganglia [27,28]. Further, from ganglia via retrograde axonal transport, herpesviruses can reach their target cells in the CNS [29,30]. This route may be supplemented by another plausible pathway via the vascular system where viruses can pass through the blood-brain barrier (BBB) to invade the brain parenchyma [12,31]. 

Previous studies of HHV-6, -7 have suggested their neurotropic nature and broad neural cell tropism in vitro and in vivo [32,33,34,35,36,37]. Bending happens due to cell membrane cofactor protein CD46 which is expressed on almost all cell types’ envelopes, viral glycoproteins that can form heterodimeric complexes to facilitate attachment and entry in the host cell, and host cell membrane proteins [38,39,40,41,42,43,44]. Furthermore, both HHV-6A and HHV-6B species have neuropathogenic pothential and capability to infect different neural cell lines [1,45,46].

Despite current knowledge, neither the most affected brain areas nor the role of HHV-6, -7 in the development of neurological disorders has been fully clarified [47]. The functional brain tissue deficit is one of the features of biological aging as well, and the etiology of this deficiency and its association with human beta-herpesviruses is still being studied.

Herein, we evaluated possible associations between immunoexpression of HHV-6, -7, and cellular immune response across different brain regions. The study aimed to explore and compare HHV-6, -7 infection within the gray and white matter of frontal and temporal lobes in cases of unspecified encephalopathy vs nonpathological conditions. We tested for the presence of viral DNA by nPCR and viral antigens by immunohistochemistry.

## 2. Results

### 2.1. Nested and Real Time Polymerase Chain Reactions

As a first step, both HHV-6 and -7 DNA were detected in 56.3% (27/48) of tissue samples from all unspecified encephalopathy cases (UEP) (Table 1). Controls revealed 33.3% (16/48) samples with both HHV-6 and -7 DNA. In both UEP group and controls HHV-6B was detected.

Further, in the UEP group, HHV-6 DNA was detected in 37.5% (9/24) and HHV-7 DNA – 16.7% (4/24) of the frontal lobe tissue samples. Concurrent HHV-6, -7 DNA was detected in two frontal lobe tissue samples of this group. In the temporal lobe, HHV-6 DNA was detected in 45.8% (11/24) and HHV-7 DNA in 25% (6/24) of UEP cases. Concurrent HHV-6 and HHV-7 infection was detected just in one temporal lobe tissue sample of this group. 

In the control group, both HHV-6 and HHV-7 virus-specific sequences were revealed in 16.7% (4/24) of the frontal lobe tissue samples, but in the temporal lobe, HHV-6 DNA was detected in 29.2% (7/24), and HHV-7 DNA in 16.7% (4/24) of analysed samples. Concurrent HHV-6 and HHV-7 infection was detected in three samples of this group. HHV-6 and HHV-7 loads (> 10 copies/10^6^ cells) were considered as elevated (PCR+).

### 2.2. Immunohistochemistry

**Figure 1:**
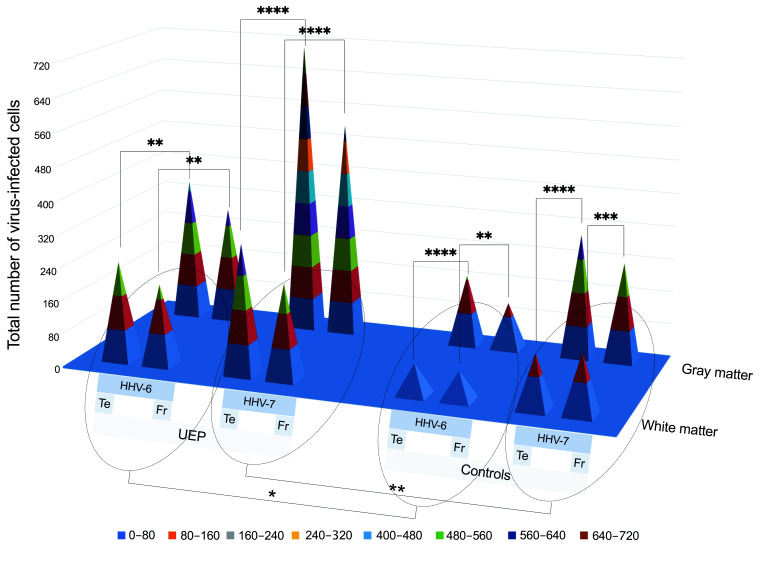
Pyramidal 3D surface plot represents the data of brain tissue immunohistochemical (IHC) analysis: total numbers of herpesvirus -6, -7 (HHV-6, -7) positive cells in the white and gray matter of the unspecified encephalopathy (UEP) group and controls. The blue plane represents a zero level (no virus-infected cells in the visual fields). Asterisks represent a significance level between different groups (* *p* < 0.05, ** *p* < 0.01, *** *p* < 0.001, **** *p* < 0.0001; chi-square test for proportions).

There was a small number of HHV positive neurons in both UEP group and controls. Higher numbers of HHV-6, -7 positive cells were found in the frontal and temporal gray matter compared to the white matter in both UEP group and controls; moreover, the highest number of HHV immunopositive cells was detected in the temporal lobe (Figure 1, Table 2).

**Figure 2:**
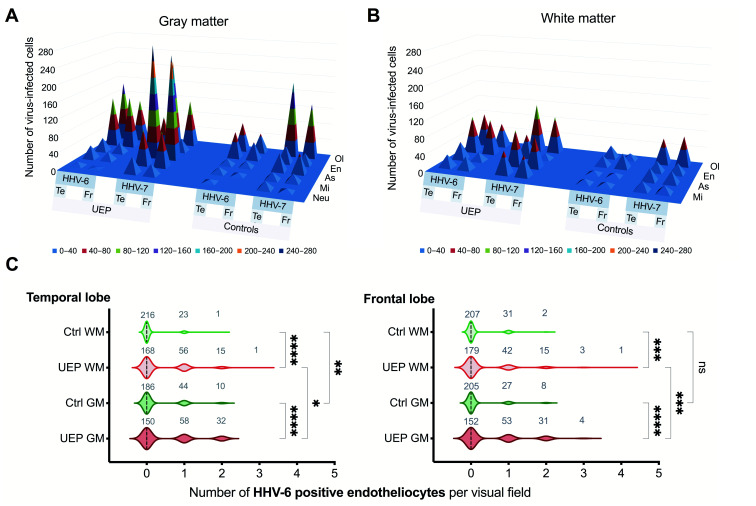
Pyramidal 3D surface and violin plots representing the data of brain tissue immunohistochemical (IHC) analysis: (**A**) total numbers of herpesvirus-6, -7 (HHV-6, -7) positive Neu (neurons), As (astrocytes), Ol (oligodendrocytes), Mi (microglia), En (endotheliocytes) in the gray matter of the unspecified encephalopathy (UEP) group and controls, frontal lobe (FR), temporal lobe (Te); (**B**) total numbers of HHV-6, -7 positive As (astrocytes), Ol (oligodendrocytes), Mi (microglia), En (endotheliocytes) in the white matter of the UEP group and controls, frontal lobe (FR), temporal lobe (Te); (**C**) distribution of total HHV-6 immunopositive endothelial cells per visual fields in the white (WM) and gray matter (GM) of the control (Ctrl) and UEP group in the temporal and frontal lobe. The blue plane represents a zero level (no virus-infected cells in the visual fields). Violin plots: dashed lines represent the approximate ratio of visual fields (out of 240) with HHV-6 positive endotheliocytes to fields with HHV negative cells (“0”— ratio less than 1.0, “1”— more than 1.0); numbers in gray show visual fields; asterisks represent a significance level (* *p* < 0.05, ** *p* < 0.01, *** *p* < 0.001, **** *p* < 0.0001) between group differences (Kruskal-Wallis test ).

In comparison with controls in both studied regions, the total number of HHV-6 positive glial cells in the UEP group’s gray and white matter was significantly higher (*p* < 0.001). Further, there were significantly (*p* < 0.001) more HHV-6 positive oligodendrocytes in the gray matter when compared to the white matter of given areas in the UEP group and controls. A significantly (*p* < 0.001) increased number of HHV-6 positive endotheliocytes was found especially in the gray matter of both frontal and temporal lobes in the UEP group in comparison with controls (Table 2, Figure 2). 

The total numbers of HHV-7 positive cells were significantly increased (*p* < 0.001) in the gray and white matter of the temporal lobe in comparison with the frontal lobe of the UEP group, also when compared to HHV-7 immunopositivity found in the controls (Figure 2). A significant difference (*p* < 0.001) was found between the HHV-7 positive oligodendrocytes located in the temporal and frontal gray matter of the UEP group. In the UEP group, significantly (*p* < 0.001) higher numbers of HHV-7 positive astrocytes were found in the gray matter of the temporal and frontal lobes in comparison with the white matter, respectively (Table 2).

**Figure 3:**
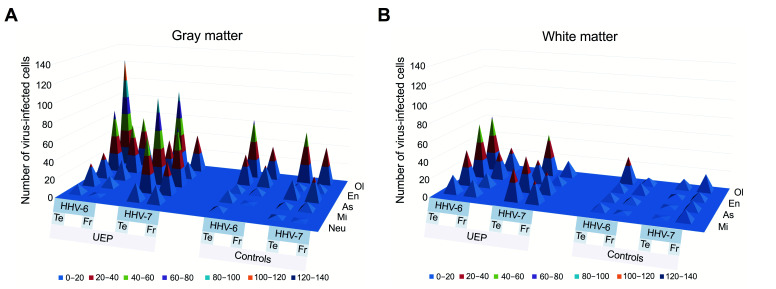
Pyramidal 3D surface plots representing the data of immunohistochemical (IHC) analysis in the PCR+ samples: numbers of herpesvirus-6, -7 (HHV-6, -7) positive Neu (neurons), As (astrocytes), Ol (oligodendrocytes), Mi (microglia), En (endotheliocytes) in the gray (**A**) and white (**B**) matter of the unspecified encephalopathy (UEP) group and controls, frontal lobe (FR), temporal lobe (Te). The blue plane represents a zero level (no virus-infected cells in the visual fields).

Furthermore, based on nested polymerase chain reaction (nPCR) results, HHV positive tissue samples (PCR+) were analyzed using immunohistochemistry (IHC) (Table 3, Figure 3).

**Figure 4:**
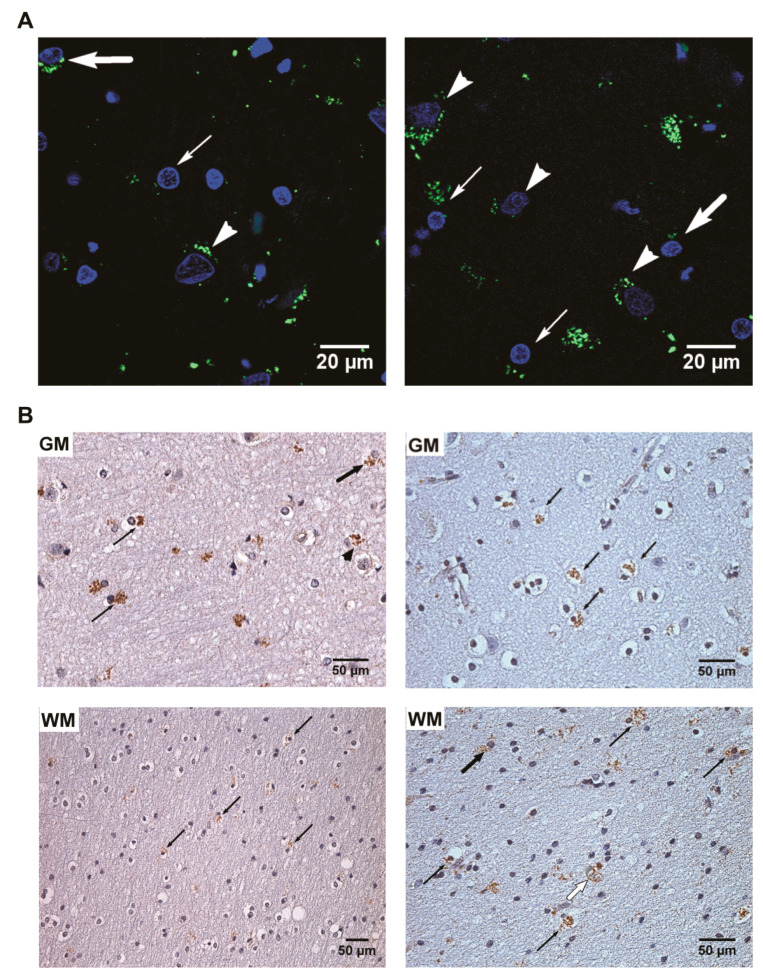
The presence of herpesvirus-6 (HHV-6) positive cells in the PCR+ samples: (**A**) detection of HHV-6 antigens by immunofluorescence, confocal microscopy (1000×), DAPI—blue, HHV-6 immunopositive products—green. Left: frontal gray matter—HHV-6 positive neuron (arrowhead), astrocyte (thick arrow), oligodendrocyte (narrow arrow) of the unspecified encephalopathy (UEP) subject; right: temporal gray matter—HHV-6 positive neuron (arrowhead), astrocyte (thick arrow), oligodendrocytes (narrow arrow) of the UEP subject; (**B**) detection of HHV-6 antigens by routine immunohistochemistry (IHC), HHV-6 immunopositive products—brown. Left: frontal gray (GM) and white (WM) matter of the UEP subject, neuron (arrowhead), astrocytes (thick arrow), oligodendrocytes (narrow arrow), (400×, 250×); right: temporal gray (GM) and white (WM) matter of the UEP subject, astrocytes (black thick arrow), oligodendrocytes (black narrow arrow), endotheliocytes (white thick arrow), (400×, 400×).

In the PCR+ samples, IHC results showed an increased number (*p* < 0.001) of HHV-6 positive oligodendrocytes in the gray matter of the frontal lobe in the UEP group when compared to controls. An increased number (*p* < 0.001) of HHV-6 positive astrocytes in the white matter of the temporal lobe in the UEP group was found when compared to controls. The UEP group revealed no significantly increased HHV-6 positive endothelial cells in the gray and white matter of the frontal lobe in comparison with controls, while in the temporal lobe, an increased number of HHV-6 positive endothelial cells was found in the white matter (Figure 4).

**Figure 5:**
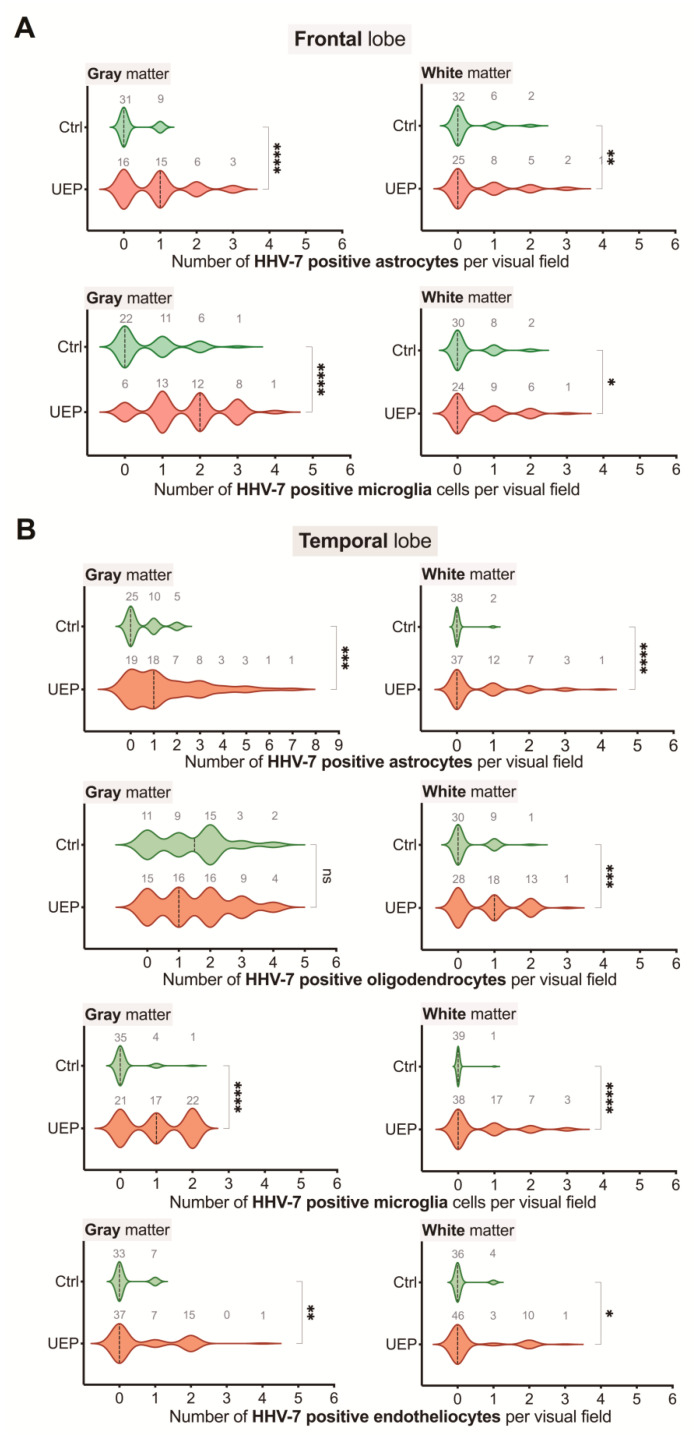
Violin plots representing the data of herpesvirus-7 (HHV-7) analysis in the PCR+ samples: (**A**) distribution of HHV-7 positive cells per visual fields in the gray and white matter of the control (Ctrl) and unspecified encephalopathy (UEP) group, frontal lobe; (**B**) distribution of HHV-7 positive cells per visual fields in the gray and white matter of the control (Ctrl) and UEP group, temporal lobe; violin plots with dashed lines representing the approximate ratio of respective visual fields with HHV positive cells to fields with HHV negative cells (“0”— ratio less than 1.0, “1”— more than 1.0); numbers in gray show visual fields; asterisks represent a significance level (* *p* < 0.05, ** *p* < 0.01, *** *p* < 0.001, **** *p* < 0.0001; Mann-Whitney U-test).

**Figure 6:**
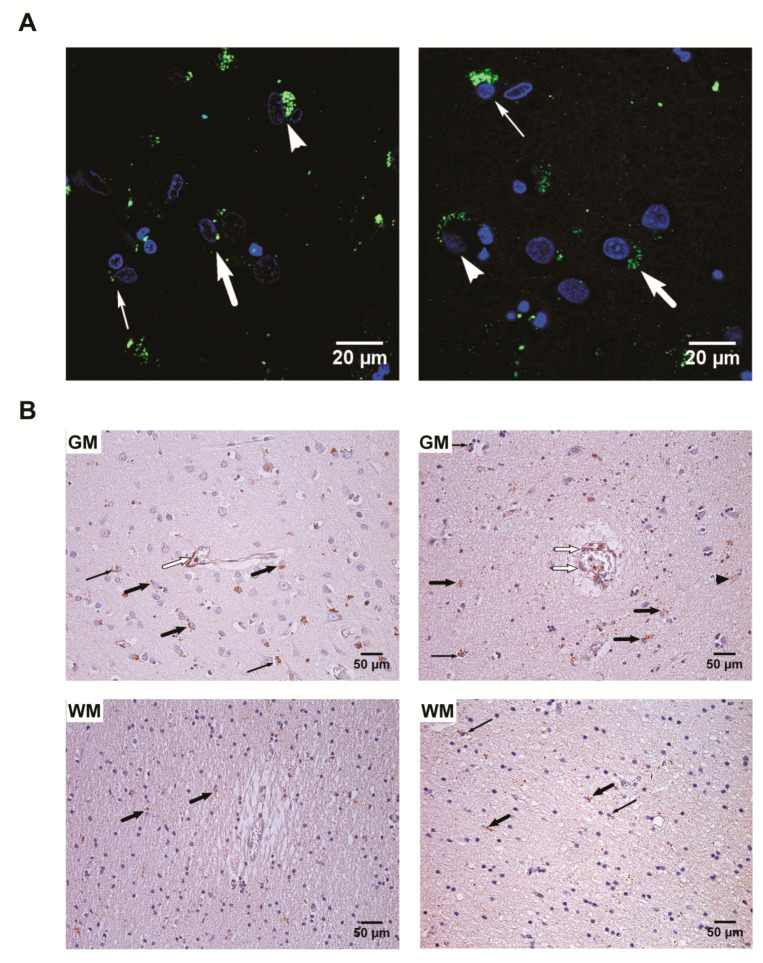
The presence of herpesvirus-7 (HHV-7) positive cells in the PCR+ samples: (**A**) detection of HHV-7 antigens by immunofluorescence, confocal microscopy (1000×), DAPI—blue, HHV-7 immunopositive products—green. Left: frontal gray matter—HHV-7 positive neuron (arrowhead), astrocyte (thick arrow), oligodendrocyte (narrow arrow) of the unspecified encephalopathy (UEP) subject; right: temporal gray matter—HHV-7 positive neuron (arrowhead), astrocyte (thick arrow), oligodendrocyte (narrow arrow) of the UEP subject; **(B**) detection of HHV-7 antigens by routine immunohistochemistry (IHC), HHV-7 immunopositive products—brown. Left: frontal gray (GM) and white (WM) matter of the UEP subject, astrocytes (black thick arrow), oligodendrocytes (black narrow arrow), vascular bed with positive mononuclear cell in the lumen (white thick arrow), (250×, 250×); right: temporal gray (GM) and white (WM) matter of the UEP subject, neuron (arrowhead), astrocytes (black thick arrow), oligodendrocytes (black narrow arrow), vascular bed with positive mononuclear cell in the lumen (white thick arrow), (250×, 250×).

In the PCR+ samples, IHC results showed increased numbers (*p* < 0.001) of HHV-7 positive astrocytes and microglia in the gray and white matter of the frontal lobe in the UEP group when compared to controls. The UEP group revealed a significantly increased (*p* < 0.001) number of HHV-7 positive glial cells in the gray matter of the temporal lobe with the most prominent positivity within astrocytes and oligodendrocytes ( Figure 5; Figure 6).

An increased total number (*p* < 0.001) of CD68 positive cells was detected in the white matter of the frontal and temporal lobes of the UEP group in comparison with controls (Table 4). Similarly, in the control cases, the placement of CD68 positive cells was more in the white matter (*p* < 0.001) in comparison with gray matter. 

In the UEP group and control cases, significantly more (*p* < 0.001) CD68 positive cells with the diffuse arrangement in the white matter of both lobes were found. A similar observation was made in the gray matter except for the UEP frontal lobe, wherein a significantly increased (*p* < 0.001) number of perivascular CD68 positive cells was detected. 

It is interesting that a significantly high (*p* < 0.001) increase in the number of CD68 positive cells was detected in the UEP temporal gray matter in comparison with the frontal lobe. 

Furthermore, based on nPCR results, the presence and location of CD68 positive cells were analyzed in the PCR+ (both HHV-6 and HHV-7) and PCR negative (PCR-) samples (Table 5).

**Figure 7:**
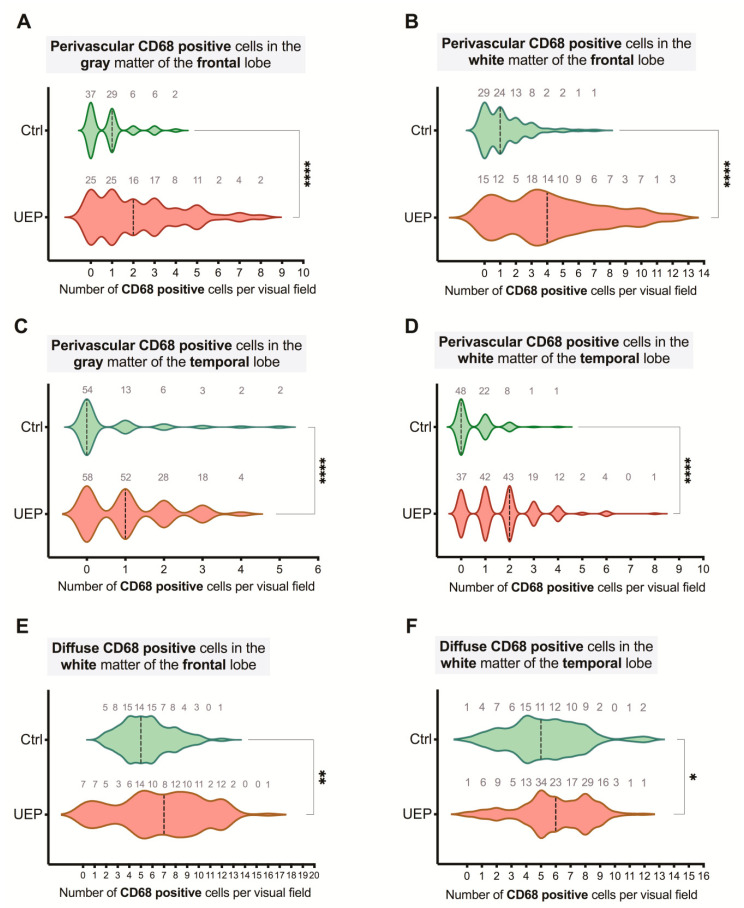
Violin plots representing the data of CD68 analysis: perivascular (**A**–**D**) and diffuse (**E**,**F**) distribution of CD68 positive cells per visual fields in the gray and white matter of the unspecified encephalopathy (UEP) group and controls (Ctrl) in the PCR+ brain samples, frontal lobe temporal lobe; violin plots: dashed lines represent the approximate ratio of respective visual fields with HHV positive cells to fields with HHV negative cells (“0”— ratio less than 1.0, “1”— more than 1.0); asterisks represent a significance level (* *p* < 0.05; ** *p* < 0.01; **** *p* < 0.0001; Mann-Whitney U-test).

**Figure 8:**
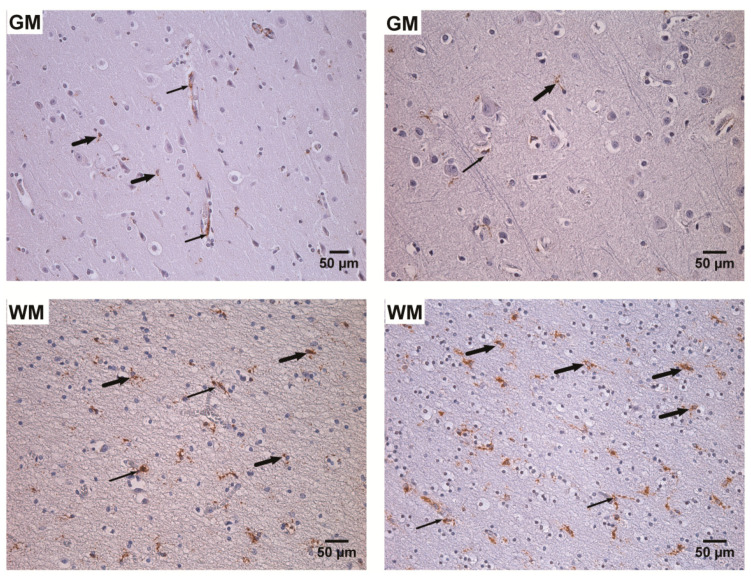
Detection of CD68 positive cells by routine immunohistochemistry (IHC), CD68 immunopositive products—brown. Left: frontal gray (GM) and white (WM) matter of the unspecified encephalopathy (UEP) subject demonstrating brown reaction products in the activated microglia/macrophages (perivascular (thin arrows) and diffuse (thick arrows) location), (200×, 250×); right: temporal gray (GM) and white (WM) matter of the UEP subject demonstrating brown reaction products in the activated microglia/macrophages (perivascular (thin arrows) and diffuse (thick arrows) location), (250×, 250×).

The highest total activated microglia/ macrophage numbers of the PCR+ UEP samples in the temporal lobe were detected. In the HHV+ cases of the UEP group, an increased number of diffuse located CD68 positive cells in the temporal areas, especially in the white matter was detected (Table 5). In the HHV+ cases of the control group, an increased number of diffuse located activated microglia/ macrophages in the gray matter was detected in comparison with HHV- control cases, where the more perivascular organization of these cells was found ( Figure 7 and Figure 8). 

There was a negligible number of lymphocytes in the white and gray matter of the UEP and control groups. Due to the low numbers of perivascular CD4 and CD8 positive cells, no significant associations between the pathological changes and HHV presence in all included cases were found.

## 3. Discussion

Since the discovery of human beta-herpesviruses and their ability to persist lifelong in their hosts, augmented interest in the possible role of the virus in development of neurodegenerative and mood diseases has been shown [6,15,16,47]. Our study used autopsy materials of frontal and temporal cortical and subcortical areas of 48 individuals with and without (controls) diagnosis of unspecified encephalopathy. 

By using combined methods it is possible to model complex relationships between HHV-6,-7 infected brain areas, and varied cell types that serve as reservoirs for the virus within them. Initially, in our study viral DNA was detected using the PCR technique. Further, IHC was used to determine the presence and intracellular localization of HHV-6, -7 proteins in the human brain tissue.

Our nPCR results showed that HHV-6B DNA is commonly found in brain tissue samples of individuals with UEP, as well as in the control group. Although significantly increased frequency of HHV-6 genomic sequence in comparison with the HHV-7 genomic sequence was found in the UEP group, more precise data on HHV immunolocation were obtained by the immunohistochemistry. Remarkably, a significant increase in presence of HHV antigens, especially HHV-7, was detected in the temporal gray matter of the UEP group. This observation confirms earlier study reports on HHV findings in the cerebral cortex, deep nuclei, and cerebellum [15,35,48]. Our previous study indicated more HHV-6 positive cells in the white vs gray matter of olfactory pathways [35,49]. However, also in the present study, we observed significantly higher numbers of HHV-6, -7 positive cells in the white matter of the UEP group vs controls. Although more HHV-6 positive neural cells were detected in the gray matter of the UEP group, demonstrating the heterogeneity of damage in the cortex vs subcortical white matter, however it does not preclude the involvement of virus in changes of white matter in the earlier stage [11]. Our study findings regarding increased numbers of HHV-6, -7 positive oligodendrocytes and astrocytes in the temporal lobe support the hypothesis about viral infection as the causative agent of neurodegenerative diseases [9,10]. Possibly, HHV-6, -7 contributes to the demyelination process by the affection of oligodendrocytes in the white matter. 

Despite our results suggesting that HHV-6, -7 may have a role in neurodegeneration, an alternative possibility cannot be ruled out. Defects in cellular immunity can lead to neurodegeneration, and this may result in the presence of persistent HHV-6, -7 [50,51]. The presence of the virus can indicate a particular symbiosis between the virus and the host in certain diseases [13,19]. It is not surprising to find HHV positive macrophages – monocyte related cells with high affinity and well-known tropism for beta-herpesviruses [52]. Finally, neural susceptibility to HHV-6, -7 may link to invalid cellular immune response, followed by development of persistent viral infection. 

As reviewed in detail elsewhere, the members of beta-herpesvirus subfamily can activate and/or affect peripheral blood T lymphocytes and cells of the hematopoietic lineage in vitro and in vivo [51,53,54]. Upon viral infection, immune system cells may influence the switch between the lytic and latent phase, that way providing HHV symbiotic machinery in the host organism [23]. Many studies support a role of CD8+ and CD4+ T cells, immune mediators that are responsible for inhibiting viral replication [19,55,56]. We found a low number of lymphocytes in contrast to monocytes-derived cells in the samples included in our study. An increased number of activated microglial cells/macrophages in the white matter of the frontal and temporal lobes of the UEP group vs controls was detected. Furthermore, the highest expression of CD68 in the gray and white matter of the temporal lobe in the HHV+ UEP cases was observed. It may be due to the requirement for phagocytic cells in the pathogen rich region and pathological changes in the area [26]. Microglia can exert a direct antiviral effect showing phagocytic activities in the brain, as reviewed by Chen and colleagues [25]. It is known that susceptibility to infection and the chance of reactivation of dormant infectious agents increases when host defense abilities decrease. One of the states, when this occurs, is when a person is getting older, so it was meaningful to evaluate HHV-6, -7 expression in the gray and white matter of frontal and temporal lobes of autopsy materials of the elderly. In the CNS, microglia are the resident phagocytes of the innate immune system. Traditionally, microglia is regarded as a key to the inflammatory process developing in response of nervous tissue to various harmful influences. In this case, activated microglia can produce various proinflammatory cytokines and immune mediators, thus creating a neurotoxic milieu leading to the progression of diseases.

We found notably high numbers of HHV-6 infected endothelial cells, especially in the temporal gray matter of the UEP group. Surprisingly, an increased number of HHV-7 positive vascular bed cells was found in the nPCR negative cases as well. The BBB is a highly specialized structure consisting of both endothelial cells and astrocytes. These co-players form a functional ‘neurovascular unit’ which has an essential role in the maintenance of a normal CNS function [57]. It is known that astrocyte-endothelial cell interaction influences the BBB in both physiological and pathological conditions [58]. It should be further investigated whether the endothelial cells can retain the herpesvirus by concentrating it in the vascular bed or, conversely, altered endotheliocytes can serve as a pathway for the virus to enter the brain parenchyma. 

Although we found herpesvirus-infected neurons, especially in the case of HHV-7, our observations suggest that larger cell tropism is associated with glial and endothelial cells in both UEP group and controls and in accordance to Domingues et al. review can modulate demyelination lesions [59]. As noted above, altered cell tropism to HHV found in this study does not exclude the two herpesvirus pathways. The overall effect of concurrent HHV-6 and -7 on brain tissue is still unclear, as there were few cases in our study. 

Further experimental evidence is needed to focus on cell-derived inflammatory and anti-inflammatory cytokines, bringing fundamental studies closer to clinical applicability and finding a set of biomarkers for early diagnosis of changes in brain matter and viral localization.

The main limitation of our research is the relatively low number of individuals included in the UEP and control groups. We did not succeed in finding supplementary data in the public data bases. Thus, larger groups are required in future to verify these results. In order to obtain a comprehensive information on the inflammatory reaction in tissues, it would be necessary to continue with a wider range of immunomarkers.

## 4. Materials and Methods 

### 4.1. Tissue Material and Sampling

Brain autopsy samples from 24 unspecified encephalopathy (UEP) cases and 24 age-matched cases without neuropathology (control group) were used in this study. In the UEP group, brain autopsies from 16 males and 8 females (mean age 63.5 (range 42–76)), and in the control group, age-matched 20 males and 4 females (mean age 61.4 (range 41–77)) were included. 

In the UEP group, autopsies revealed an enlarged side and third ventricles, without hemorrhagic or ischemic infarctions in the brain matter and without hemorrhagic changes in the meninges. In the control group, pathomorphological unchanged brain autopsies without dilated ventricles, hemorrhagic or ischemic infarctions in the brain matter and without meningeal hemorrhagic changes were collected [60]. In the medical histories, no clinical features were found regarding brain/neurological pathologies.

Cohorts of UEP and controls were de-identified after the death and before the conventional autopsies. Tissue samples were obtained at the Department of Pathology, Riga 1st hospital, and the Latvian State Centre for Forensic Medical Examination. The *post mortem* range between 7 and 30 hours was respected. Protocols for obtaining postmortem brain tissue complied with all institutional guidelines with special respect for identity confidentiality.

Within the framework of Latvian Council of Science Grant Nr.478/2012, the study protocol and the use of brain tissue autopsy samples were approved by the Ethics Committee of Rīga Stradiņš University (Decision of the RSU Ethics Committee No. 30/05/2013) on 30 May 2013. 

### 4.2. Nested and Real Time Polymerase Chain Reactions

Nested polymerase chain reaction (nPCR) for the qualitative detection of HHV-6, -7 genomic sequences in DNA isolated from fresh frontal and temporal lobe samples was used. Total DNA was isolated from tissue samples using standard phenol-chloroform extraction. To ensure the quality of extracted DNA, a β-globin PCR was performed. PCR amplification for the HHV-6, -7 was conducted using 1 µg of the tissue DNA [60]. 

Primers complementary to the U3 gene that encodes main capsid proteins for both HHV-6A and HHV-6B and U10 gene for HHV-7 were used. Positive controls (HHV-6 and HHV-7 genomic DNA; ABI, Columbia, MD, USA) and negative controls (DNA obtained from practically healthy HHV-6 and HHV-7 negative blood donors), as well as water controls were included in each experiment. 

Presence of HHV-6 U3 gene sequence was detected using the following primers: Cycle 1: HV1 forward-5’- GCGTTTTCAGTGTGTAGTTCGGCAG- 3’

HV2 reverse- 5’- TGGCCGCATTCGTACAGATACGGAGG- 3’

Cycle 2: HV3 forward- 5’- GCTAGAACGTATTTGCTGCAGAACG- 3’

HV4 reverse- 5’- ATCCGAAACAACTGTCTGACTGGCA- 3’

Presence of HHV-6 LTP gene sequence was detected with the following primers:

Cycle 1: O1 - 5′- AGTCATCACGATCGGCGTGCTATC- 3′ 

O2 - 5′-TATCTAGCGCAATCGCTATGTCG-3′

Cycle 2: I3 - 5′-TCGACTCTCACCCTACTGAACGAG- 3′

I4 - 5′-TGACTAGAGAGCGACAAATTGGAG- 3′

Obtained nPCR amplification products were digested with HindIII restriction endonuclease (Thermo Scientific, USA) which cleaves HHV-6B 163 bp amplification product into 66 bp and 97 bp fragments, whereas does not cleave HHV-6A.

Presence of HHV-7 U10 gene sequence was detected using the following primers: 

Cycle 1: HV7 forward- 5’ – TATCCCAGCTGTTTTCATATAGTAAC – 3’ 

HV8 reverse- 5’ – GCCTTGCGGTAGCACTAGATTTTTTG – 3’ 

Cycle 2: HV10 forward- 5’ – CAGAAATGATAGACAGATGTTGG – 3’ 

HV11 reverse- 5’ – TAGATTTTTTGAAAAAGATTTAATAAC – 3’

Real-Time PCR with the β-globine gene as an internal control was performed. HHV-6 load was determined with HHV-6 Real-TM Quant (Sacace Biotechnologies, Italy). The test contains an IC (β-globine gene), which serves as an amplification control for each individually processed specimen and to identify possible reaction inhibition.

HHV-7 load was detected using REALQUALITY RS-HHV 7 kit (AB ANALITICA Advanced biomedicine, Padua, Italy) with the β-globine gene as an internal control or using Human Herpes Virus 7 genomes genesig kit (Primerdesign, Eastleigh, United Kingdom), also with an internal control. 

HHV-6 and HHV-7 loads (> 10 copies/10^6^ cells) were considered as elevated. Detection of HHV-6 and -7 DNA was done following Secchiero and Berneman et al. [61,62]. 

### 4.3. Immunohistochemistry

Anti-HHV-6 (20) mouse monoclonal IgG, raised against viral lysate for immunohistochemical (IHC) detection of HHV-6A and HHV-6B (Santa Cruz Biotechnology, Inc., Santa Cruz, CA, USA, 1:200), anti-HHV-7 antibody raised against the tegument protein pp85 of HHV-7 (Advanced Biotechnologies, Columbia, MD, USA, 1:500) were used for detection of virusspecific antigene expression, and CD68 mouse monoclonal antibody (Cell Marque, Rocklin, CA, USA, clone Kp-1, 1:200) for detection of activated microglia/macrophages in brain tissue samples. For quantitative analysis of immune system cells in the autopsy material, anti CD4 (Cell Marque, Rocklin, CA, USA, clone SP35, 1:100) and anti CD8 (Cell Marque, Rocklin, CA, USA, clone C8/144B, 1:100) mouse monoclonal antibodies were used. The myelin basic protein (MBP, Santa Cruz Biotechnology, Inc., Santa Cruz, CA, USA, clone 1.B.645, 1:150) and glial fibrillary acidic protein (GFAP, Novocastra, Leica Biosystems, Newcastle, UK, clone GA5, 1:100) was used to detect oligodendrocytes and astrocytes, respectively. For all the reactions negative controls were performed replacing the primary antibodies with a PBS solution. For positive controls, tissue samples from osteoarthritis and rheumatoid arthritis cases with HHV+ detected by nPCR, and with known antibody positivity were used. Representative figures are added in the supplement (Figure S1).

Brain tissues were prepared in accordance with the standard histopathology protocol, followed by immunohistochemistry and fluorescence microscopy as described previously [63]. Paraffin-embedded 4–5 µm histological sections were deparaffinized and hydrated in xylene and series of graded ethanol, respectively. The activity of endogenous peroxidase was reduced by 30% hydrogen peroxide in methanol (30 min). Antigen retrieval was performed in 0.01 M citrate buffer (15 min) at 96 °C. Following the manufacturer’s recommendations, sections were incubated overnight (4 °C) with the primary antibodies. HiDef Detection™ HRP Polymer system (CellMarque, Rocklin, CA, USA) was used for visualization of antigen–antibody complexes. Sections were successively incubated with HiDef Detection™ Amplifier for 10 min (RT) and HiDef Detection™ HRP Polymer Detector for 10 min (RT) after rinsing in phosphate-buffered saline (PBS). The antigens were visualized by 3,3’ diaminobenzidine (DAB) tetrahydrochloride kit (DAB+Chromogen and DAB+Substrate buffer, Cell Marque, Rocklin, CA, USA) for 5 min. Thereafter, sections were stained by Mayer’s hematoxylin, rinsed with tap water, dehydrated, cleared, and embedded in Roti® Histokitt (Carl Roth, Karlsruhe, Germany).

Antigen expression was assessed quantitatively by counting the number of immunopositive cells. 

Expression of antigens was estimated in 10 randomly selected vision fields of each sample at ×400 magnification using a Leica light microscope (LEICA, LEITZ DMRB, Germany) and Glissando Slide Scanner (Objective Imaging Ltd., Cambridge, UK). Duplicable measurements of tissue markers were obtained, including gray and white matter of the frontal and temporal lobes using Aperio ImageScope program v12.2.2.5015 (Leica Biosystems, Buffalo Grove, IL, USA).

For immunofluorescence, after immunostaining with the primary antibody, sections were washed with PBS buffer (3 × 5 min), and then with secondary goat anti-mouse IgG (H+L) antibody, Alexa Fluor® 488 conjugate (Thermo Fisher Scientific, Invitrogen, UK, 1:300) was applied. The tissue staining with 4′,6-diamidino-2-phenylindole (DAPI) (Thermo Fisher Scientific, Invitrogen, UK, 1:3000) was performed to analyze the arrangement of cell nuclei. After that, sections were embedded in Prolong Gold with DAPI (Thermo Fisher Scientific, Invitrogen, UK). Before the coverslipping, the autofluorescence effect was minimized using 0.2% Sudan Black B solution (Sigma Aldrich, St. Louis, MO, USA). All immunofluorescence images were captured using a Nikon confocal microscope Eclipse Ti-E (Nikon, Brighton, MI, USA). 

### 4.4. Data Analysis

Numerical data distribution of nPCR and IHC results was analysed by the D’Agostino and Pearson, Anderson–Darling, and Shapiro–Wilk normality tests. Different groups of numerical variables were compared by one-way ANOVA or one-way ANOVA on ranks and Kruskal–Wallis test followed by a two-stage step-up method of Benjamini, Krieger, and Yekutieli as a post hoc test. Brown-Forsythe and Bartlett’s tests were applied for testing the homogeneity of variances. For categorical variables, the chi-square test was performed. For the comparison of numerical values between two groups, the two-tailed Mann-Whitney U test was applied. IHC results are expressed as violin plots, and a p-value less than 0.05 (*p* < 0.05) was considered statistically significant. In violin plots, the medians (visualized as dashed lines) were used to represent the approximate ratio of visual fields (max out of 240) with HHV-positive cells to fields with HHV-negative cells (“0” – ratio less than 1.0, “1” – more than 1.0). 

All the graphs, calculations, and statistical analyses were performed using the program GraphPad Prism 9 (GraphPad Software, La Jolla, CA, USA). 

## 5. Conclusions

Our results suggest that HHV-6 and -7 may have a role in the brain micro-environment. Or perhaps, an alternative possibility is if brain parenchyma is characterized by defects in cellular immunity, it may lead to the persistence of HHV-6 and/or -7 in some kinds of brain cells.

HHV-6, predominantly located in oligodendrocytes, and HHV-7, predominantly located in astrocytes and oligodendrocytes, exhibit greatly varying effects on neural homeostasis, although there is no evidence of viral replication. A high number of activated microglia observed in the temporal areas in the UEP group serves as an indicator of the cellular immune response to HHV. The HHV antigens found in endotheliocytes suggest that BBB is involved in the spread of HHV. Analysis of HHV-positive cell distribution reveals a causal role of the virus in healthy and changed conditions. The question remains of whether the human beta-herpesviruses contribute to the neurological diseases or are markers for some aspect of the disease process. Because of the conflicting results in the medical literature regarding the role of HHV-6 and HHV-7 infection in the development of neurologic diseases, further research should be performed to confirm or deny the direct causality of neurologic disorders due to herpesvirus -6, -7 in the human brain. 

## Figures and Tables

**Table 1:** The presence of single herpesvirus-6, 7 (HHV-6, -7) genomic sequences and HHV-6, -7 co-infection in tissue DNA samples of the frontal and temporal lobes in the unspecified encephalopathy (UEP) individuals and control group.

Group	Lobe		Samples Containing Viral Genomic Sequences (n)				
		*n*	HHV-6 (a)	HHV-7 (b)	HHV-6 + HHV-7 (c)	Total ((a+b)-c)	p-Value (Chi^2^) (vs. Control)
UEP	Frontal	24	9	4	2	11	0.3759
	Temporal	24	11	6	1	16	0.0209
Controls	Frontal	24	4	4	0	8	
	Temporal	24	7	4	3	8	

**Table 2:** Numbers of herpesvirus-6, -7 (HHV-6, -7) immunopositive cells in tissue samples of the frontal and temporal lobes in the UEP individuals and control group.

**Groups**	**Lobes**	Viral Genomic Sequence	Gray Matter						White Matter				
			Neu (*n*)	Mi (*n*)	As (*n*)	En (*n*)	Ol (*n*)	Total (*n*)	Mi (*n*)	As (*n*)	En (*n*)	Ol (*n*)	Total (*n*)
UEP	Te	HHV-6	10	32	33	122	148	345	30	42	89	79	240
	Fr	HHV-6	2	21	26	127	105	281	31	21	85	58	195
	Te	HHV-7	39	86	278	85	218	706	55	86	57	116	314
	Fr	HHV-7	27	87	257	33	115	519	49	76	15	89	229
Controls	Te	HHV-6	7	15	15	64	70	171	11	9	25	30	75
	Fr	HHV-6	0	7	15	43	47	112	10	9	35	15	69
	Te	HHV-7	10	18	42	44	184	298	12	30	26	61	129
	Fr	HHV-7	8	20	48	29	134	239	18	29	26	71	144

Legend: UEP—unspecified encephalopathy, Fr—frontal lobe, Te—temporal lobe, Neu—neurons, Mi—microglia, As—astrocytes, En—endotheliocytes, Ol—oligodendrocytes, n—a number of HHV-6 and HHV-7 positive cells in the given areas.

**Table 3:** Numbers of herpesvirus-6, -7 (HHV-6, -7) immunopositive cells in the PCR+ tissue samples of the frontal and temporal lobes in the UEP individuals and control group.

**Groups**	**Lobes**	Viral Genomic Sequence	Gray Matter						White Matter				
			Neu (*n*)	Mi (*n*)	As (*n*)	En (*n*)	OL (*n*)	Total (*n*)	Mi (*n*)	As (*n*)	En (*n*)	Ol (*n*)	Total (*n*)
UEP	Te	HHV-6	10	24	28	70	124	256	20	39	61	63	183
	Fr	HHV-6	2	12	21	55	56	146	17	15	31	27	90
	Te	HHV-7	19	61	96	41	91	308	35	39	26	47	147
	Fr	HHV-7	19	36	66	18	41	180	24	24	10	18	76
Controls	Te	HHV-6	5	12	9	33	65	124	8	9	10	30	57
	Fr	HHV-6	0	1	7	23	36	67	2	7	12	8	29
	Te	HHV-7	3	6	20	7	56	92	1	2	4	11	18
	Fr	HHV-7	1	9	26	9	41	86	12	10	9	19	50

Legend: UEP—unspecified encephalopathy, Fr—frontal lobe, Te—temporal lobe, Neu—neurons, Mi—microglia, As—astrocytes, En—endotheliocytes, Ol—oligodendrocytes, *n*—a number of HHV-6 and HHV-7 positive cells in the given areas.

**Table 4:** An arrangement of total CD68 positive cells in samples of the frontal and temporal lobes of UEP individuals and control group.

Group	Lobe	Gray Matter			White Matter		
		Diffuse (*n*)	Perivascular (*n*)	Total (*n*)	Diffuse (*n*)	Perivascular (*n*)	Total (*n*)
UEP	Fr	314	466	780	1458	864	2322
	Te	431	245	676	1289	376	1665
Controls	Fr	273	193	466	901	323	1224
	Te	284	185	469	842	233	1075

Legend: UEP—unspecified encephalopathy, Fr—frontal lobe, Te—temporal lobe, *n*– a number of CD68 positive cells in the given areas.

**Table 5:** An arrangement of the total number of CD68 positive cells in the frontal and temporal lobes of UEP individuals and control group comparing herpesvirus (HHV) PCR+ with HHV PCR- tissue samples.

Group	Lobe	Gray Matter			White Matter		
		Diffuse (*n*)	Perivascular (*n*)	Total (*n*)	Diffuse (*n*)	Perivascular (*n*)	Total (*n*)
UEP	Fr_HHV+	188	251	439	740	478	1218
	Fr_HHV-	126	215	341	718	386	1104
	Te_HHV+	325	178	503	947	275	1222
	Te_HHV-	106	67	173	342	101	443
Controls	Fr_HHV+	142	67	209	445	105	550
	Fr_HHV-	131	126	257	456	218	674
	Te_HHV+	170	58	228	418	42	460
	Te_HHV-	114	127	241	424	191	615

Legend: UEP—unspecified encephalopathy, Fr—frontal lobe, Te—temporal lobe, Fr_HHV+/T_HHV+ – HHV-6 and/or HHV-7 nPCR positive sample, Fr_HHV-/T_HHV- – HHV-6 and/or HHV-7 nPCR negative sample, *n*– a number of CD68 positive cells in the given areas.

## Supplementary Materials

The following are available online at https://www.mdpi.com/1422-0067/22/5/2364/s1.
