# Supplementary material for: Human Herpesvirus-6 and -7 in the Brain Microenvironment of Persons with Neurological Pathology and Healthy People
Source: Int J Mol Sci. 2021 Feb 27;22(5):2364. doi: 10.3390/ijms22052364 (PMC7956495; doi:10.3390/ijms22052364)
Supplement: Supplementary file 1 [file ijms-22-02364-s001.pdf]

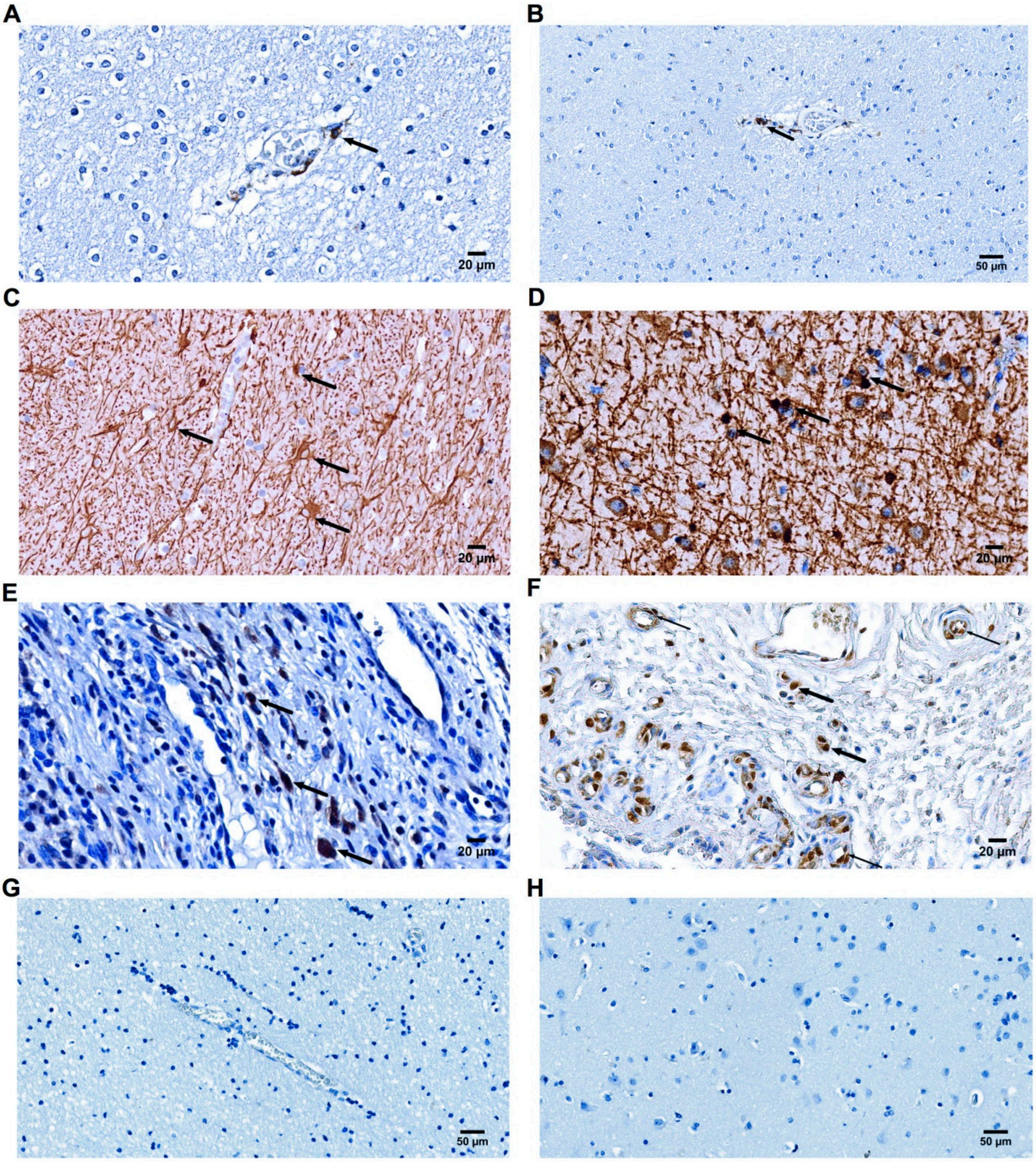

Figure S1. Representative microphotographs of IHC. (**A**) CD4 immunoreactivity observed in the temporal white matter of HHV+ individual (arrow), ×400; (**B**) CD8 immunoreactivity observed in the frontal white matter of HHV+ individual (arrow), ×200; (**C**) GFAP immunopositivity in the astrocytes (arrows), temporal gray matter, ×400; (**D**) MBP immunopositivity in the oligodendrocytes (arrows), temporal gray matter, ×400. (**E**) Immunoreactivity observed in the synovial stroma of HHV-6+ subject with an osteoarthritis: HHV-6+ macrophages (arrows), ×400. (**F**) Immunoreactivity observed in the synovial stroma of HHV-7+ subject with a rheumatoid arthritis: HHV-7+ macrophages (thick arrows) and vascular endotheliocytes (narrow arrows), ×400. (**G**) HHV-6 negative control, frontal white matter, ×200; (**H**) HHV-7 negative control, temporal gray matter, ×200.
